# Pulmonary tuberculosis with cutaneous manifestations

**DOI:** 10.6026/973206300220989

**Published:** 2026-02-28

**Authors:** Preetam Singh Katroliya, Pradeep Singh Rajoriya, Rohit S Shukla, Neerja Kain, Rajesh Kumar Ahirwar

**Affiliations:** 1 Department of Skin and Venereal Diseases, SRVS Medical College, Shivpuri, Madhya Pradesh, India; 2 Departments of Respiratory Medicine, SRVS Medical College, Shivpuri, Madhya Pradesh, India; 3 Departments of Respiratory Medicine, Gajra Raja Medical College, Gwalior, Madhya Pradesh, India; 4 Consultant pathologist, District hospital, Shivpuri, Madhya Pradesh, India; 5 Departments of Community Medicine, SRVS Medical College, Shivpuri, Madhya Pradesh, India

**Keywords:** Pulmonary tuberculosis (PTB), cutaneous tuberculosis, lupus vulgaris, scrofuloderma, extrapulmonary TB

## Abstract

Pulmonary tuberculosis (PTB) rarely presents with cutaneous manifestations, which can pose significant diagnostic and management challenges for healthcare providers. In this hospital-based study of 123 confirmed PTB patients, 18 (14.6%) showed cutaneous involvement, predominantly lupus vulgaris (33.3%) and scrofuloderma (27.8%). Cutaneous manifestations were significantly associated with previous TB treatment, extrapulmonary involvement and severe radiological disease. Lupus vulgaris and scrofuloderma were most frequently linked with active pulmonary tuberculosis. This study advances knowledge by emphasizing the clinical relevance of cutaneous signs as markers of disease burden and the need for integrated dermatological and pulmonary assessment.

## Background:

Pulmonary tuberculosis (TB) remains a leading cause of morbidity and mortality worldwide, with over 10 million new cases reported annually [1]. Extrapulmonary manifestations are noted in approximately 15-20% of TB patients and cutaneous involvement, though rare, can present critical diagnostic challenges [2]. Cutaneous tuberculosis (CTB) accounts for roughly 1-1.5% of extrapulmonary TB cases, with forms such as lupus vulgaris, scrofuloderma, tuberculosis verrucosa cutis and tuberculous gumma representing classic presentations [3]. CTB exhibits diverse clinical phenotypes, ranging from plaques and nodules to ulcers and abscesses, often mimicking other dermatologic conditions and leading to misdiagnosis and treatment delay [4]. In regions with high TB prevalence and among immunocompromised individuals, atypical morphologies-including panniculitis-like lesions and ulcerative presentations have been increasingly reported [5]. Diagnosis of CTB requires a high index of suspicion, particularly in patients with concurrent pulmonary disease. Histopathological evaluation, microbiological culture and molecular techniques such as PCR on skin biopsies are pivotal for confirmation [6]. Standard anti-tubercular therapy remains effective in most CTB cases, though prolonged courses and adjunctive treatments may be warranted based on lesion type and immune status [7]. Therefore, it is of interest to explore the clinical spectrum of CTB in patients with pulmonary TB, emphasizing diagnostic pitfalls, classification and management strategies to enhance early detection and improve outcomes.

## Methodology:

This study was conducted as a hospital-based observational study in an Indian tertiary care teaching medical college. Patients of all age groups presenting with pulmonary tuberculosis (PTB), confirmed microbiologically or radiologically, who also exhibited cutaneous manifestations, were included. Patients with known immunosuppressive disorders, HIV infection, or those unwilling to participate were excluded. A total of 123 patients fulfilling the inclusion criteria were recruited by consecutive sampling during the study period. Diagnosis of pulmonary tuberculosis was established using sputum smear microscopy, cartridge-based nucleic acid amplification test (CBNAAT), chest radiography and where necessary, culture for Mycobacterium tuberculosis. Cutaneous tuberculosis was diagnosed based on clinical examination by a dermatologist, supported by skin biopsy, histopathology and microbiological confirmation where available. Demographic details, clinical history, duration of symptoms, past history of tuberculosis and treatment status were recorded using a structured proforma. Detailed dermatological examination was performed to document type, distribution and morphology of skin lesions. Data were entered into Microsoft Excel and analyzed using SPSS software version 26.0. Categorical variables were presented as frequencies and percentages, while continuous variables were summarized as mean ± standard deviation. Associations between clinical variables and cutaneous manifestations were assessed using chi-square or Fisher's exact test. A p-value <0.05 was considered statistically significant.

## Results:

The study included 123 patients with pulmonary tuberculosis, of whom 18 (14.6%) presented with cutaneous manifestations. The mean age of the cohort was 42.7 ± 13.5 years and there was no significant age difference between patients with and without cutaneous involvement (p = 0.62). Males accounted for 63.4% of the study population, with a comparable distribution across both groups (p = 0.74). Residence, smoking, alcohol use, HIV co-infection and diabetes showed no statistically significant association with cutaneous disease, although HIV co-infection was relatively more frequent among those with skin lesions (22.2% vs. 7.6%, p = 0.08) (Table 1). With regard to disease-related characteristics, patients with cutaneous tuberculosis were significantly more likely to have a history of previous TB treatment (33.3% vs. 12.4%, p = 0.02) and extrapulmonary TB (38.9% vs. 15.2%, p = 0.03). In addition, severe radiological disease was more common in those with cutaneous manifestations (55.6% vs. 30.5%, p = 0.04). Other variables, including prolonged symptom duration and multidrug-resistant TB, did not differ significantly between the two groups (Table 2). Among the 18 patients with cutaneous manifestations, the most common lesion was lupus vulgaris (33.3%), followed by scrofuloderma (27.8%), tuberculosis verrucosa cutis (16.7%), papulonecrotic tuberculid (11.1%) and erythema induratum (11.1%) (Table 3).

## Discussion:

Cutaneous tuberculosis (CTB) is a rare manifestation of extrapulmonary tuberculosis but remains clinically significant. In our study, CTB was observed in 14.6% of patients with pulmonary tuberculosis, higher than commonly reported prevalence rates [8]. This higher rate may reflect regional variation, referral patterns, or heightened clinical vigilance. The most frequent lesions were lupus vulgaris (33.3%) and scrofuloderma (27.8%), consistent with other reports on cutaneous tuberculosis in endemic regions These lesions typically result from hematogenous or lymphatic dissemination of Mycobacterium tuberculosis from pulmonary foci. We observed a significant association of CTB with previous TB treatment (p = 0.02), extrapulmonary involvement (p = 0.03) and severe pulmonary disease on radiography (p = 0.04), suggesting that cutaneous involvement may indicate more advanced or disseminated tuberculosis. This emphasizes the importance of comprehensive evaluation in patients with skin lesions [9]. Demographic variables such as age, sex, smoking, alcohol use, HIV co-infection and diabetes mellitus were not significantly associated with CTB in our cohort, in contrast to some prior studies that suggested these as risk factors [10]. This discrepancy may reflect sample size limitations or population differences. Clinically, CTB lesions are polymorphic and can mimic other dermatologic conditions, which may delay diagnosis. Lupus vulgaris and scrofuloderma are often chronic, slowly progressive and require histopathological confirmation for definitive diagnosis [11]. Early recognition of cutaneous lesions can therefore serve as a diagnostic clue and help in identifying patients with more severe systemic disease [12].

## Conclusion:

Cutaneous manifestations were seen in a notable number of pulmonary tuberculosis patients, most commonly lupus vulgaris and scrofuloderma. Although less frequent than pulmonary involvement, skin lesions reflect systemic disease and were significantly associated with prior TB treatment, extrapulmonary involvement and severe pulmonary disease. Recognizing these lesions through careful dermatological evaluation can aid diagnosis, indicate disease severity and support comprehensive patient management.

## Figures and Tables

**Table 1:** Demographic and clinical profile of patients with pulmonary tuberculosis (n = 123)

**Variable**	**Total (n=123)**	**With Cutaneous Manifestations (n=18, 14.6%)**	**Without Cutaneous Manifestations (n=105, 85.4%)**	**p-value**
Age (years)	42.7 ± 13.5	44.1 ± 12.9	42.4 ± 13.7	0.62
Male sex (%)	78 (63.4%)	12 (66.7%)	66 (62.9%)	0.74
Rural residence (%)	69 (56.1%)	11 (61.1%)	58 (55.2%)	0.65
Smoking history (%)	41 (33.3%)	9 (50.0%)	32 (30.5%)	0.11
Alcohol use (%)	28 (22.8%)	6 (33.3%)	22 (21.0%)	0.21
HIV co-infection (%)	12 (9.8%)	4 (22.2%)	8 (7.6%)	0.08
Diabetes mellitus (%)	17 (13.8%)	3 (16.7%)	14 (13.3%)	0.71

**Table 2:** Disease-related characteristics and association with cutaneous manifestations

**Variable**	**Total (n=123)**	**With Cutaneous Manifestations (n=18)**	**Without Cutaneous Manifestations (n=105)**	**p-value**
Duration of TB symptoms >6 mo	31 (25.2%)	7 (38.9%)	24 (22.9%)	0.14
Previous TB treatment (%)	19 (15.4%)	6 (33.3%)	13 (12.4%)	0.02*
Extrapulmonary TB (%)	23 (18.7%)	7 (38.9%)	16 (15.2%)	0.03*
MDR-TB (%)	9 (7.3%)	3 (16.7%)	6 (5.7%)	0.11
Severe radiological disease (%)	42 (34.1%)	10 (55.6%)	32 (30.5%)	0.04*
*p < 0.05 = statistically significant

**Table 3:** Types of cutaneous manifestations observed (n=18)

**Type of Lesion**	**n (%)**
Lupus vulgaris	6 (33.3%)
Scrofuloderma	5 (27.8%)
Tuberculosis verrucosa cutis	3 (16.7%)
Papulonecrotic tuberculid	2 (11.1%)
Erythema induratum	2 (11.1%)
